# Basal Cell Ameloblastoma of Mandible: A Rare Case Report with Review

**DOI:** 10.1155/2013/187820

**Published:** 2013-04-08

**Authors:** Hemant Shakya, Vikram Khare, Nilesh Pardhe, Ena Mathur, Mansi Chouhan

**Affiliations:** ^1^Department of Oral Medicine and Radiology, Mahatma Gandhi Dental College & Hospital, Jaipur 302022, Rajasthan, India; ^2^Department of Oral & Maxillofacial Pathology, Mahatma Gandhi Dental College & Hospital, Jaipur 302022, Rajasthan, India

## Abstract

Ameloblastoma is a slow-growing benign neoplasm that has a strong tendency to local invasion and that can grow to be quite large without metastasizing. Rare examples of distant metastasis of an ameloblastoma in lungs or regional lymph nodes do exist. It has an aggressive and recurrent course and is rarely metastatic. Radiographically it shares common features with other lesions such as the giant cell tumor, aneurysmal bone cyst, and renal cell carcinoma metastasis; a definitive diagnosis can only be made with histopathology. Basal cell ameloblastoma is believed to be the rarest histologic subtype in which the tumor is composed of more primitive cells and has even fewer features of peripheral palisading. Till date, only few cases of basal cell ameloblastoma have been reported in the literature. Considering the rarity of the lesion, we report here an interesting and unique case of basal cell ameloblastoma of the mandible occurring in a very old patient.

## 1. Introduction

Ameloblastomas are benign locally aggressive, polymorphic neoplasms of proliferating odontogenic epithelial origin, arising from cell rests of enamel organ, either remnants of dental lamina or remnants of Hertwig's sheaths, the epithelial rest of Malassez, the developing enamel organ, basal cell epithelium of the jaws, heterotropic epithelium in the other parts of the body especially the pituitary gland and epithelium of the odontogenic cyst particularly the dentigerous cyst, and the odontomas [1]. Ameloblastomas constitute approximately 1% of all cysts and tumors of the jaws. The occurrence in the mandible is five times higher than in the maxilla. In the literature, the average age of occurrences is 38.9 years [2].

The incidence, clinical and radiological features, behavior, and histopathology of ameloblastomas have been extensively reviewed in numerous publications. They occur in three different clinicoradiographic situations, which deserve separate consideration because of different therapeutic considerations and prognosis [3].

These are

1. conventional solid or multicystic (about 86% of all cases),
2. unicystic (about 13% of all cases),
3. peripheral (extraosseous) (about 1% of all cases).

Several histopathologic patterns of ameloblastomas are commonly described and include the follicular, plexiform, acanthomatous, granular cell, and basal cell patterns. There appears to be rather general agreement that these variations in histopathology patterns do not have any significant bearing on prognosis except unicystic ameloblastoma because of the less aggressive behavior and favorable prognosis [4].

Basal cell variant of ameloblastoma is the least common type. These lesions are composed of nests of uniform baseloid cells and histopathologically very similar to basal cell carcinoma of skin. No stellate reticulum is present in the centre portion of the nest. The peripheral cells around the nest tend to be cuboidal rather than columnar [3].

Though very little information appears in the literature either due to insufficient number of cases reported or due to variations in the clinical or radiological criteria, the pathologist may sometimes fail to differentiate it from intraoral basal cell carcinoma [4].

The purpose of this paper is to present a case of the rarest variant of ameloblastoma (basal cell ameloblastoma) that has occurred in the 6th decade of the age and to provide a brief review of the literature.

## 2. Case Report

**Figure 1:**
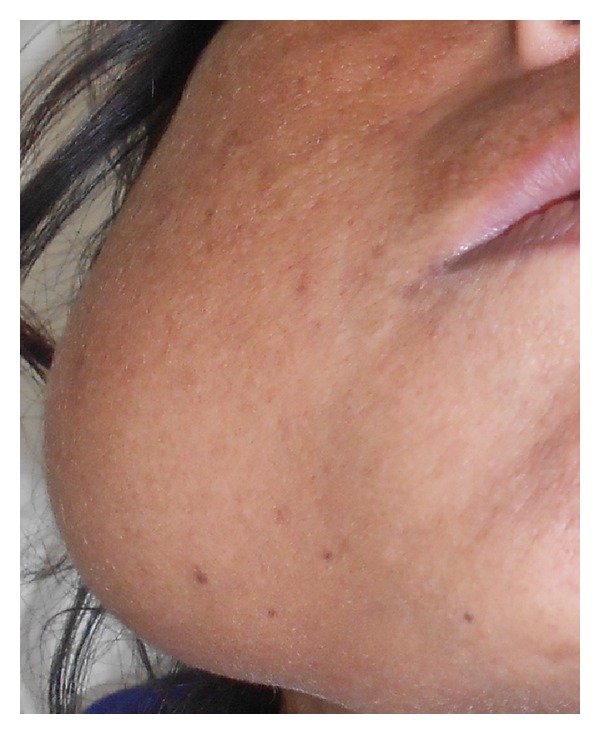
Frontal view of the patient showing extraoral swelling in the right lower mandibular region.

**Figure 2:**
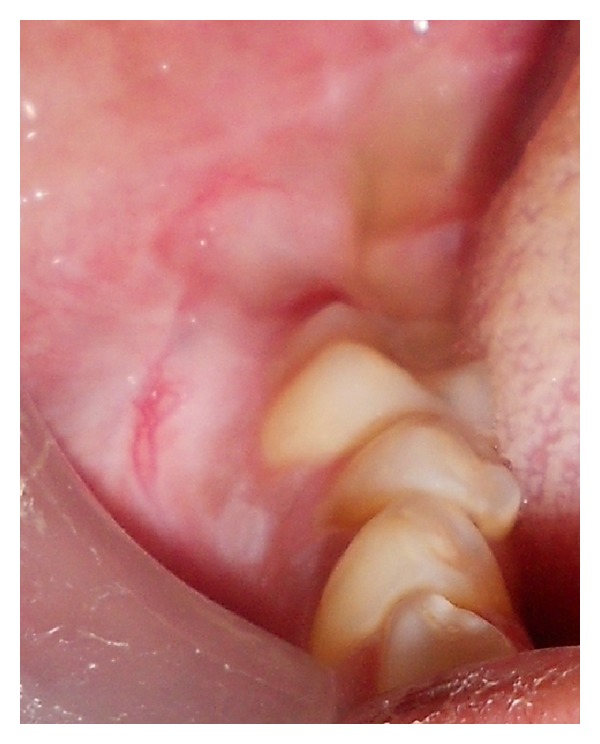
Intraoral view of the patient showing obliteration of the right lower buccal vestibule.

A 50-year-old female patient visited the department of oral medicine and radiology, with a chief complaint of the pain and swelling over the right lower third of the face for 6 months. Extraorally swelling (Figure 1) was oval in shape, size 7 cm × 6 cm and extended anteriorly from the right corner of the oral cavity to posterior border of ramus of mandible. Superiorly from right tragus of ear to 1 cm below to the lower border of mandible. Intraoral examination (Figure 2) revealed obliteration of the right lower buccal vestibule. The overlying skin of lesion was normal in color without any sinus and drainage, and there was no local rise in the temperature. The patient had poor oral hygiene and numbness of the lower lip on right side. On the basis of history and clinical examination the provisional diagnosis was given as odontogenic tumur and differential diagnosis was given as odontogenic cyst and bone tumur. 

**Figure 3:**
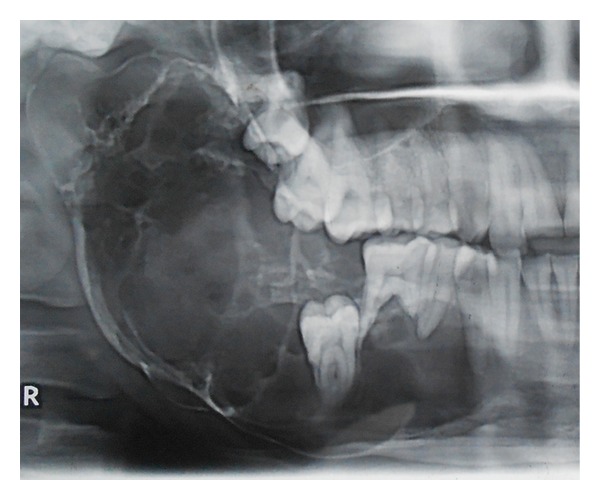
OPG of the female patient showing multilocular radiolucent lesion & root resorption of 46.

**Figure 4:**
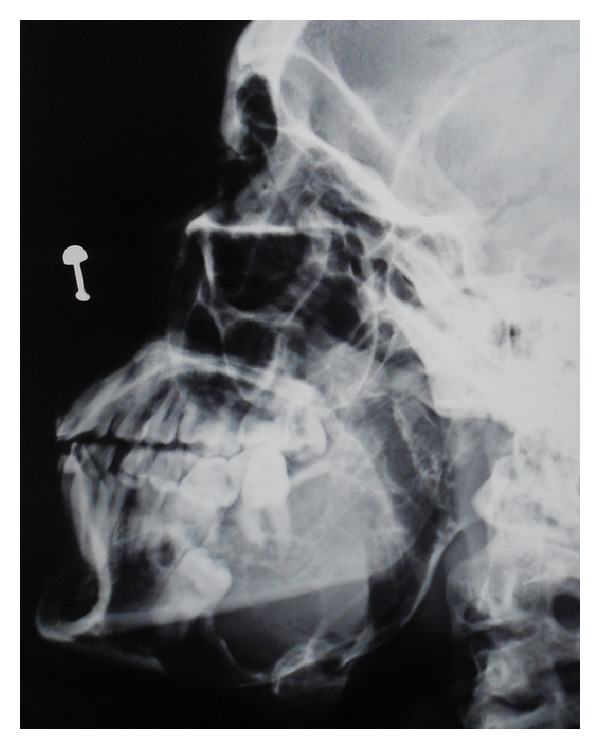
Lateral cephalogram of the patient showing expansion of the lower border of mandible.

**Figure 5:**
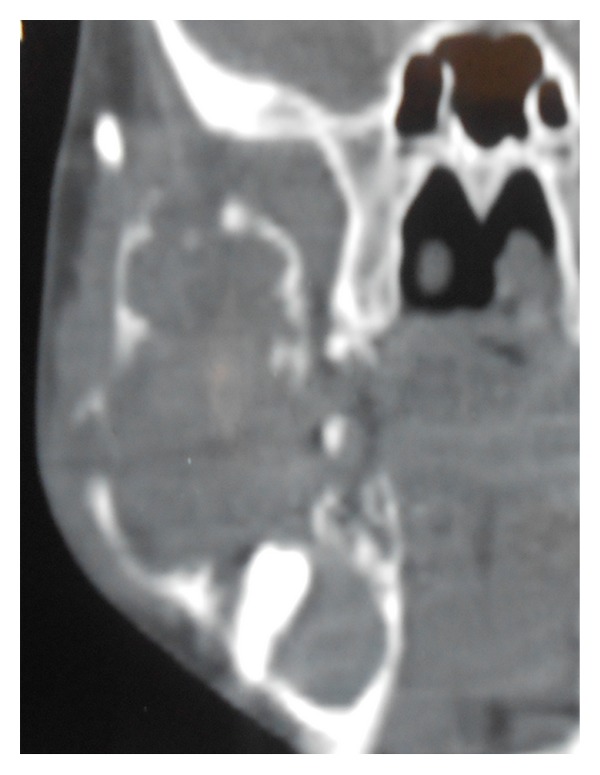
CT scan of the patient showing bicortical expansion with perforation.

The details of the procedure were explained to the patient and a written informed consent was obtained. The patient was subjected to routine hematological and radiological examination. Radiographically in orthopentomogram (Figure 3) lesion was multilocular and extended mesially from canine to condylar and coronoid region. The panoramic view revealed expansion of lower border of mandible & anterior border of ramus with perforation. Root resorption of 46 was in favour of ameloblastoma. Expansion of lower border of mandible is evident in lateral cephalogram (Figure 4). CT scan (Figure 5) revealed bicortical expansion with perforation.

Incisional biopsy was taken from the site of lesion which shows features of basal cell ameloblastoma. Then the patient was refered to the department of oral and maxillofacial surgery where she underwent hemimandibulectomy and reconstruction was done with a 2.5 mm reconstruction plate; bony reconstruction was not considered due to the advanced age of the patient.

**Figure 6:**
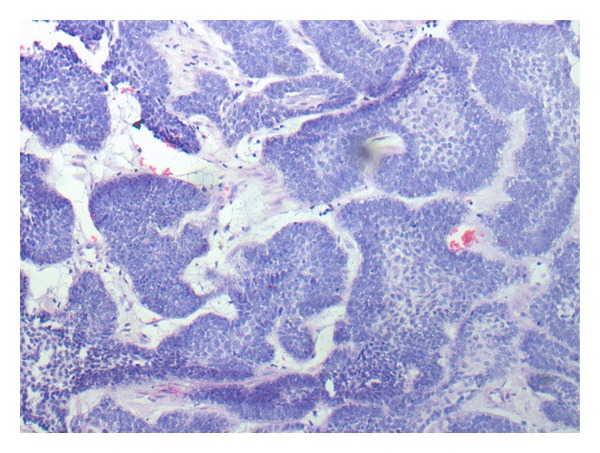
Photomicrograph shows island of uniform baseloid cells in a mature fibrous connective tissue stroma.

In the histopathology (Figure 6) report, a completely resected lesion showed islands of uniform baseloid cells in a mature fibrous connective tissue stroma. Peripheral cells are columnar & showed reversal of polarity. The cells were stained deeply basophilic and nearly equivalent in staining intensity. In some island, the central portion is completely replaced by baseloid cells and some island shows scanty stellate reticulum like areas. Baseloid cells are hyperchromatic and there is no palisading of nuclei. Healing was uneventful and she was discharged after 7 days.

## 3. Discussion

Ameloblastomas begin as unilocular lesions and evolve into multilocular lesions, according to the fact that the mean age of patient with unilocular lesion is 26 years, whereas it is 38 years for multilocular ameloblastomas. Radiographically it may appear unilocular or multilocular with soap bubble or honeycomb appearance; buccal and lingual expansion of the cortex invariably accompanies ameloblastoma. Thinned and intact cortex shows egg shell appearance [5].

Worth divided the ameloblastoma into four radiological manifestation categories.

1. First, resembling a dentigerous cyst without septa, mostly in ramus region. Most frequently anterior wall of ramus lost.
2. Second, most common, cystic appearing cavity with distinctive septa (caricature of spider). Perforation in anterior surface of ramus & superior border of the body of mandible. Characteristically, angle of mandible is preserved. The inferior aspect may be ballooned out with a significant smooth downward convexity.
3. Third, less common multilocular cystic appearance in posterior portion of mandible and ramus. Significant downward enlargement of inferior border of mandible, which maintains a convex lower border.
4. Fourth, solid varity in which normal bone is replaced by honeycomb appearance in which cavities are relatively small and fairly uniform in size.

The basal cell ameloblastoma is a rare variant of ameloblastoma, which shows a remarkable resemblance to the basal cell carcinoma and published cases of intraoral basal cell carcinoma most likely are basal cell ameloblastoma; only few cases of basal cell subtypes were available for valid statistical analysis. Basal cell ameloblastoma tends to grow in an island-like pattern. The characteristic color gradation in other ameloblastoma is often difficult to appreciate in basal cell type, because baseloid appearing cell rather then stellate reticulum-like appearing cell occupies the center portion of the tumor island. The baseloid cells stain deeply basophilic and equivalent in staining intensity with peripheral layer of cells [6].

The typical cellular morphology and nuclear orientation of peripheral cells are altered. They tend to be low columnar to cuboidal and often do not show reverse nuclear polarity with subnuclear vacuole formation, but hyperchromatism and palisading of nuclei are maintained.

The similarity in the histopathology of this tumor to basal cell carcinoma might point toward the aggressiveness of this lesion. The recurrence rates of basal cell ameloblastoma have not been reported due to the limitation in the number of cases. Basal cell variant like the one reported in the paper needs an appropriate diagnosis based not only on clinical and radiological principles but also on sound histopathologic analysis. Long-term followup is necessary to establish the recurrence rate.

## References

[1] Cherrick HennryM, Laskin DanielM (2009). Odontogenic tumors of the jaw. *Oral and Maxillofacial Surgery*.

[2] Small IA, Waldron CA (1995). Ameloblastoma of the Jaws. *Journal of Oral and Maxillofacial Surgeryand Pathology*.

[3] Neville BW, Damn DD, Allen CM, Bouqout JK (2002). *Oral and Maxillofacial Pathology*.

[4] Desai H, Sood R, Shah R, Cawda J, Pandya H (2006). Desmoplastic ameloblastoma: report of a unique case and review of literature. *Indian Journal of Dental Research*.

[5] Langland RP, Langland OE, Nortje CJ (1995). *Diagnostic Imaging of the Jaw*.

[6] Kessler HP (2004). Intraosseous ameloblastoma. *Oral and Maxillofacial Surgery Clinics of North America*.

